# Utilizing a culture system for horizontal cells to study neural circuit assembly in the developing mouse retina

**DOI:** 10.3389/fncel.2026.1691122

**Published:** 2026-04-01

**Authors:** Ross M. Perez, Yong H. Park, Ajeet Singh, Collin Todora, Marlon F. Mattos, Daniela Becerril, Rajashree Venkatraman, Arlene A. Hirano, Nicholas C. Brecha, Rinki Ratnapriya, Benjamin J. Frankfort, Elizabeth Zuniga-Sanchez

**Affiliations:** 1 Department of Ophthalmology, Baylor College of Medicine, Houston, TX, United States; 2 Department of Neurobiology, David Geffen School of Medicine at UCLA, Los Angeles, CA, United States; 3 The Veterans Administration Greater Los Angeles Healthcare System, Los Angeles, CA, United States; 4 Department of Biochemistry and Molecular Pharmacology, Baylor College of Medicine, Houston, TX, United States; 5 Department of Neuroscience, Baylor College of Medicine, Houston, TX, United States

**Keywords:** cell culture, development, horizontal cells, neurite outgrowth, retina

## Abstract

The precise wiring of the nervous system relies on neurons extending their processes at the right time and place to find their appropriate synaptic partner. The mechanisms that determine when and where neurons extend their neurites during synaptogenesis remains a central question in the field. In the present study, we used a cell culture system coupled with live imaging to investigate the wiring mechanisms in the developing mouse retina. We focused on horizontal cells which are a class of interneurons in the outer mouse retina known to synapse selectively to the distinct types of photoreceptors. Previous research has shown horizontal cells extend their neurites and make connections to their respective photoreceptor partner in a temporal- and spatial-dependent manner. However, the mechanisms responsible for their selective wiring to photoreceptors during development remains poorly understood. To answer this question, we developed a horizontal cell culture system to investigate the cellular mechanisms responsible for neurite outgrowth during circuit assembly. Our data shows cultured horizontal cells extend neurites with a similar morphology as *in vivo*. Moreover, neurite extension of horizontal cells is limited to early developmental stages as young mice extend more complex processes compared to those from adolescent retinas. We also found that horizontal cells, unlike retinal ganglion cells, do not extend neurites when cultured alone and require other retinal neurons to promote neurite outgrowth. In summary, we established a horizontal cell culture system that can be used to decipher the mechanisms involved in neural circuit assembly of the mouse retina.

## 1 Introduction

**Figure 1:**
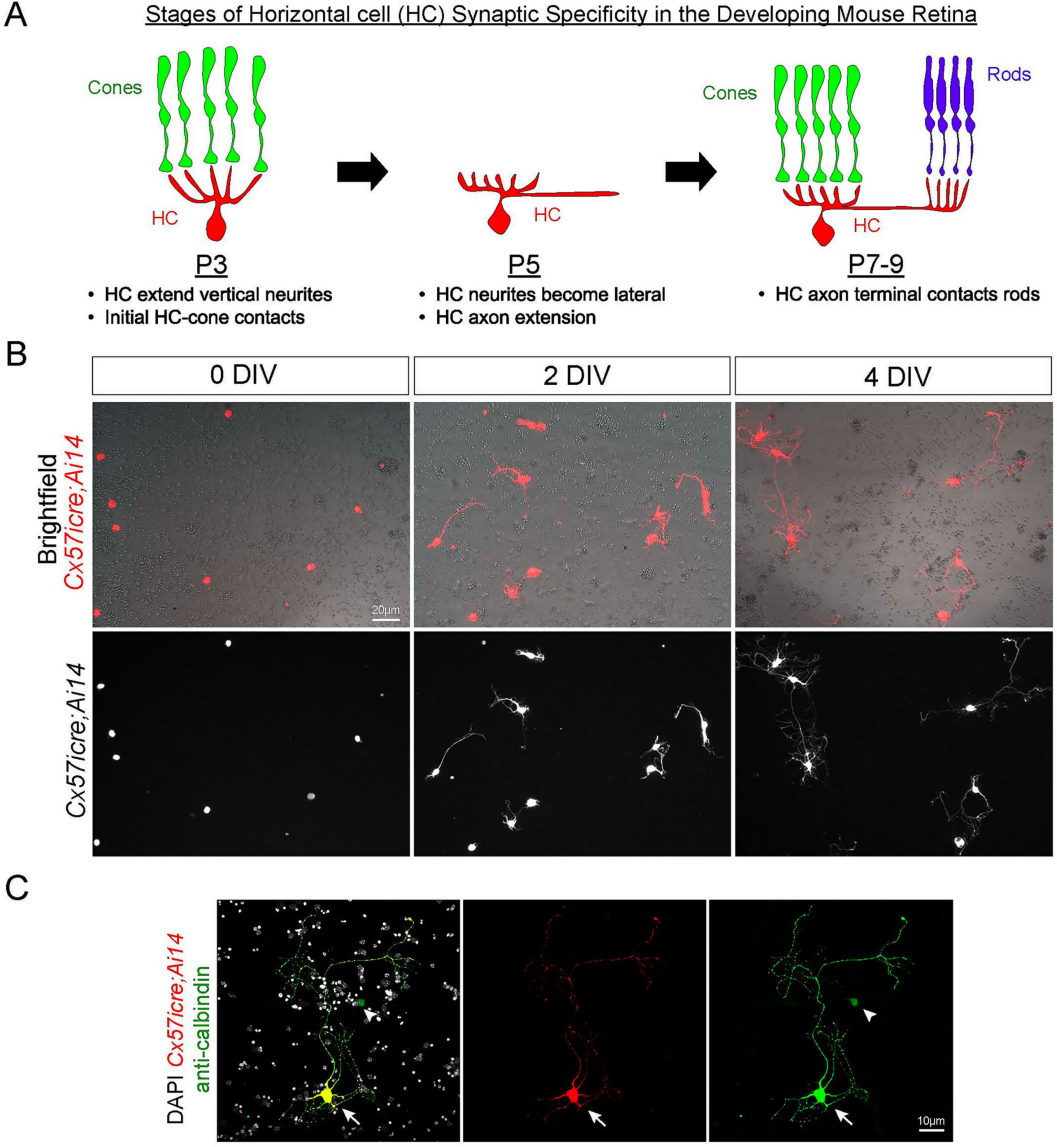
Horizontal cells extend neurites in culture with a similar morphology as *in vivo* **(A**–**C)**. Schematic drawing of the developmental stages when horizontal cells (HC) make contacts to the different types of photoreceptors (i.e., cones and rods) in mice **(A)**. Horizontal cells gradually extend neurites in culture from 0 DIV to 4 DIV **(B)**. Horizontal cells from *Tg(Cx57icre; Ai14)* animals were imaged with a 10x objective using fluorescence microscopy in **(B)**. An overlay of the brightfield and TdTomato fluorescence channel are shown in the top panel of **(B)**. The bottom panel is the TdTomato signal alone presented as a grayscale. Horizontal cells are shown in red in the top panel and white in the bottom panel **(B)**. A high-magnification fluorescent microscopy image taken with a 40x objective of a single horizontal cell at 4 DIV isolated from *Tg(Cx57icre; Ai14)* retinas is double positive for TdTomato (red) and anti-calbindin (green) as depicted by white arrow **(C)**. Calbindin also stains amacrine cells but these are not TdTomato-positive as shown by arrowhead in **(C)**.

Neural circuit assembly is an intricate and complex process where neurons make connections to distinct synaptic targets. This process is referred to as synaptic specificity [reviewed in (Sanes and Zipursky, 2020)]. To achieve synaptic specificity, neurons must extend their processes at a specific time and location to form connections to their correct target. The question of how neurons determine when and where to precisely extend their processes to find their partner remains unclear. An excellent example of synaptic specificity is found within the mouse outer retina where horizontal cells synapse selectively to the different types of photoreceptors. In the mouse retina, the dendrites of horizontal cells synapse selectively to cone photoreceptors whereas the axon terminal synapses to rod photoreceptors (Kolb, 1974). The selective wiring of horizontal cells to photoreceptors is thought to occur at various stages during development. Prior studies in mice have shown that horizontal cells first extend neurites at postnatal (P) day 3, and these neurites preferentially make contacts to cone photoreceptors (Sarin et al., 2018; Huckfeldt et al., 2009). Around P5, horizontal cells begin to extend a long process that will eventually become the axon (Veldman et al., 2020), and by P7–9, the axon terminal extends out processes to make contacts to rod photoreceptors (Olney, 1968; Rich et al., 1997; Blanks et al., 1974). See Figure 1A. These data highlight how synaptic specificity of horizontal cells is highly regulated in both a temporal and spatial manner. However, the developmental mechanisms that instruct neurite extension of horizontal cells is relatively unknown. Elucidating these mechanisms has been difficult to uncover for several reasons. First, horizontal cells as well as many other neuron types in the central nervous system overlap extensively in both their dendritic and axonal field (Kolb, 1974; Veldman et al., 2020; Soto et al., 2013). Although single labeling approaches such as DiI labeling and AAVs have been used to label single horizontal cells (Soto et al., 2013; Reese et al., 2005), performing these experiments at early developmental time points remains technically challenging. Second, live imaging of horizontal cells has been performed *in vivo* (Barrasso et al., 2018), however, this can only be done for a few hours and not days. Thus, capturing the events surrounding synaptic specificity which is known to occur from P3 to P9 is not feasible using this approach. And lastly, manipulating the environment to uncover mechanisms of horizontal cell specificity is difficult and cumbersome, heavily relying on transgenic or mutant mouse lines to alter the composition of retinal cell types (Raven et al., 2007). Thus, we developed a cell culture system for horizontal cells coupled with live imaging to overcome these challenges. To achieve this, we used the *Tg(Cx57icre)* mouse line which drives iCre expression *via* the control of the *Cx57* gene as described previously (Hirano et al., 2016). The *Cx57* gene encodes for the connexin-57 protein which is a key component of gap junctions and known to be selectively expressed in horizontal cells of the mouse retina (Hombach et al., 2004; Ciolofan et al., 2007). We thereby took advantage of the selective expression within horizontal cells using the *Tg(Cx57icre)* mouse line to begin to decipher the mechanisms underlying synaptic specificity in the wild-type mouse retina.

## 2 Materials and methods

### 2.1 Isolation and culture of horizontal cells

All methods were performed in accordance with the ARRIVE guidelines and all animal procedures were approved by the Institutional Animal Care and Use Committee (IACUC) of Baylor College of Medicine. *Tg(Cx57icre)* mice were kindly provided by Nicholas Brecha (Hirano et al., 2016), and crossed to an *Ai14* reporter mouse strain from Jackson Labs (JAX #007914) (Madisen et al., 2010). Developmental time points: P8 and P30 mice were used for all experiments. Both males and females were used indiscriminately in this study. Mice were euthanized with pentobarbital sodium (390 mg/mL) and phenytoin sodium (50 mg/mL) solution, also known as Euthasol (ANADA #200–071) with a lethal dose of 0.1 mL per mouse given via IP or a minimum of 200 mg/kg by body weight. Eyes were promptly removed after euthanasia and the entire retina was dissociated for culture with no pre-isolation steps before plating or prior to Fluorescence-activated cell sorting (FACS) using protocols modified from (Park et al., 2020). A minimum of three animals were used to generate three independent biological replicates with 3–6 technical replicates per experimental condition as listed in the figure legend. Retinas were dissected in oxygenated Ames media (#A1372-25, US Biological) and enzymatically digested with papain (#LS003126; Worthington, Lakewood, NJ) for 12 min at 37 °C followed by trituration with a P-1000 pipette. Dissociated retinal cells were then sieved through a 40 μm cell strainer (#352340; Falcon, Corning, NY) to remove clumps resulting in a single cell suspension. Following this procedure, retinal cells were spun down for 8 min in a centrifuge at 4 °C, 300 x rcf. After removal of the supernatant, retinal cells were resuspended in 4% Ames/BSA (#A-4161, Sigma-Aldrich, St. Louis, MO). Cells were then seeded at a density of 500 cells per μL onto 384-well plates (#781986; Greiner Bio-One, Monroe, SC), coated with 10°μg/mL of poly-D-lysine for one hour (#P6407; Sigma-Aldrich, St. Louis, MO, United States) and 2°μg/mL of mouse laminin l overnight (#3400–010-01; Trevigen Gaithersburg, MD). Retinal cells were cultured in Neurobasal media (#21103049; Invitrogen, Carlsbad, CA), penicillin/streptomycin (#15140–12; Gibco, Waltham, MA) with Cytosine *β*-D-arabinofuranoside (Ara-C) (5 μM) (#C6645; Sigma Aldrich, St. Louis, MO) to inhibit the growth of glial cells as described in (Park et al., 2020). For FACS, a total of six retinas from three different transgenic mice were used per experiment. The cre-mediated TdTomato fluorescence signal was used to isolate horizontal cells from the *Tg(Cx57icre; Ai14)* mouse line. Depending on the conditions, between 3–12 wells were used to plate the neurons. Each well contained 100 μL of volume. For all experiments, both male and female mice were used indiscriminately.

### 2.2 Isolation of retinal ganglion cells (RGCs)

*Vglut2-cre* mice (JAX #028863) were generously provided by Benjamin Frankfort and described previously (Pauli et al., 2022; Vong et al., 2011). *Vglut2-cre* mice were crossed with the *Ai9* TdTomato reporter mouse line (JAX #007909) (Madisen et al., 2010) to generate *Tg(Vglut2-cre; Ai9)* animals. Isolation of retinal ganglion cells (RGCs) was performed as previously described (Park et al., 2020) with minor modifications. To enrich for RGCs, immunopanning was carried out using an anti-CD73 antibody (Cat. #550738; RRID: AB_393857) to deplete rod photoreceptors. FACS was then used to collect RGCs based on CD90.2/Thy1.2 expression (Cat. #17–0902-83; RRID: AB_469422) and TdTomato fluorescence. For all experiments, both male and female mice were used.

### 2.3 Immunocytochemistry

Cell cultures were fixed in 4% paraformaldehyde for 15 min, followed by subsequent washes in PBS. Cells were then incubated with blocking buffer (10% normal goat serum, 1% BSA, 0.5% Triton X-100 in PBS) followed by overnight primary antibody incubation with Rabbit anti-calbindin (Swant Cat#CB38, RRID: AB_10000340) used at a 1:2000 dilution or Mouse anti-Rhodopsin (Abcam Cat#ab98887, RRID: AB_10696805 at a 1:500 dilution. After primary antibody incubation, cells were washed three times with PBS followed by secondary antibody incubation with either Goat anti-Rabbit-488 (Thermo Fisher Cat#A-11034, RRID: AB_2576217), Goat anti-Rabbit-647 (Thermo Fisher Cat#A-21244, RRID: AB_2535812) or Goat anti-Mouse-488 (Thermo Fisher Cat#A-11029, RRID: AB_2534088) at 1:1000 dilution at 4 °C overnight. Cells were then washed 3 times with PBS, stained with DAPI (1:1000), and then each well was filled with Vectashield (Vector Laboratories).

### 2.4 Imaging analysis

Retinal cultures were imaged using a Leica DMi8 inverted microscope (Buffalo Grove, IL) for 10x magnification images and a Zeiss LSM 800 microscope (Jena, Germany) was used to capture 40x magnification images. All quantification was performed using the 10x magnification images where 9–12 photographic fields of 1.43mm^2^ in size were taken to capture the entire well (area = 8.55 mm^2^). Timelapse videos were taken using the EVOS FL-Auto Cell Imaging System with a 20x objective (Thermo Fisher, Waltham, MA). Neurite length and cell counts were quantified using the Imaris confocal software version 9.6 (Bitplane, South Windsor, CT, United States). Total cell counts were obtained and normalized to the area measured of each well. The filament tool in Imaris was used to automatically trace individual neurons with a cell body diameter of 16 μm in size and filament thickness of 3 μm. The total filament length sum was used as the average neurite length per cell for the different experimental conditions.

### 2.5 Statistical analysis

All statistical analyses were performed in R (v4.x) using custom scripts. Technical replicates were averaged within each biological replicate, and all statistical inferences were conducted at the biological-replicate level to avoid pseudo replication. Data distributions were evaluated using the Shapiro–Wilk test on nonzero values to assess normality and guide the choice of statistical tests. When raw measurements exhibited zero inflation or strong right-skew (e.g., neurite length measurements in horizontal cells, HC-only cultures, or mixed cell-type comparisons), nonparametric tests were applied and geometric means with 95% confidence intervals (CI) were computed for nonzero subsets. For age-dependent comparisons of HC neurite length with small sample sizes (*n* = 3 biological replicates per age), plate-averaged values were log-transformed to stabilize variance and reduce the influence of zero-inflated distributions, and Welch’s *t*-test was used to accommodate unequal variance between groups. For multi-time-point co-culture experiments assessing DIV-dependent changes, omnibus testing was conducted using the Kruskal–Wallis test, followed by Dunn’s post-hoc comparisons with Benjamini-Hochberg correction for multiple testing; conditions consisting entirely of zero measurements (HC-only cultures) were excluded from statistical testing. Comparisons between HC and retinal ganglion cell (RGC) neurite outgrowth employed the Mann–Whitney U test for overall group differences, Fisher’s exact test to quantify differences in zero-inflation rates, and Wilcoxon rank-sum tests restricted to nonzero values to assess differences in neurite length independent of outgrowth. For analyses involving repeated measurements across days *in vitro* (DIV), linear repeated-measures models or paired *t*-tests with Holm correction were used, with caution applied in conditions where only two biological replicates were available due to limited statistical power. All statistical tests were two-sided unless a directional hypothesis was specified, and adjusted *p*-values < 0.05 were considered significant.

## 3 Results

### 3.1 Horizontal cells gradually extend neurites in culture similar to *in vivo* conditions

We crossed the horizontal cell-specific cre line, *Cx57icre* (Hirano et al., 2016) to the TdTomato fluorescent reporter, *Ai14* (Madisen et al., 2010) to generate *Tg(Cx57icre; Ai14)* animals. Using an optimized protocol to culture retinal neurons (Park et al., 2020), we isolated and dissociated retinas from *Tg(Cx57icre; Ai14)* mice at P8 and seeded 500 cells per μL. We found horizontal cells isolated from *Tg(Cx57icre; Ai14)* at P8 gradually extend neurites from 0 to 4 days *in vitro* (DIV) as shown in Figure 1B. We confirmed TdTomato-positive cells were indeed horizontal cells by co-staining with anti-calbindin at 4 DIV (Figure 1C). Calbindin is known to label both horizontal cells and amacrine cells (Peichl and Gonzalez-Soriano, 1994; Haverkamp and Wassle, 2000); however, the *Tg(Cx57icre)* mouse line has been reported to be specific to horizontal cells (Hirano et al., 2016; Hirano et al., 2018). Thus, only double TdTomato-positive and calbindin-positive cells were used for further data analysis. In addition, we found the Rhodopsin antibody which is known to label rod photoreceptors (Sarin et al., 2018) also worked in cell culture and appears to be specific as there is no staining within horizontal cells as shown in Supplementary Figure S1. The sequential progression of neurite outgrowth across DIV is reminiscent to what has been reported *in vivo* where horizontal cells extend neurites in a gradual and time-dependent manner.

### 3.2 Live imaging allows for visualization of cell–cell interactions

**Figure 2:**
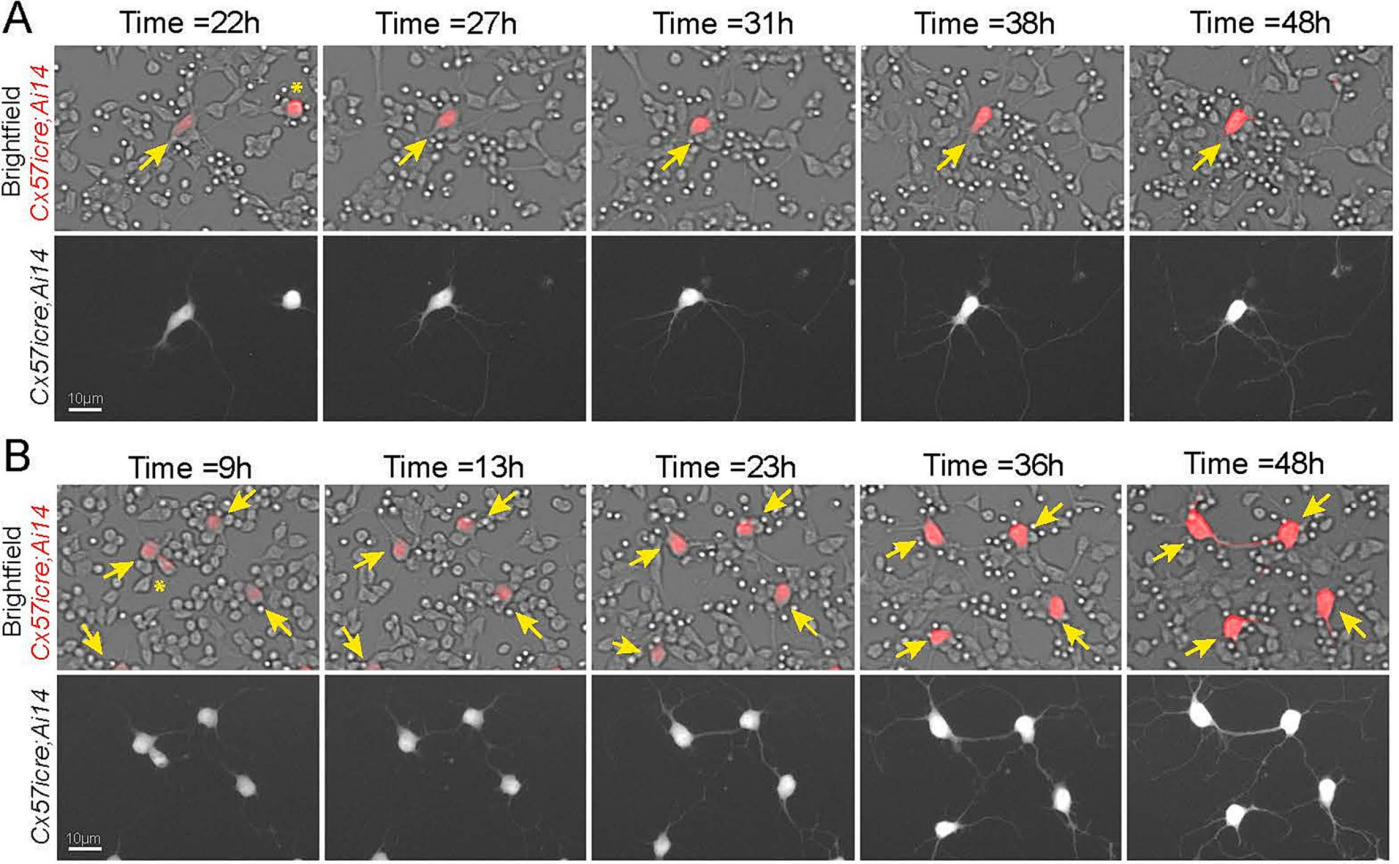
Timelapse imaging of horizontal cells in culture **(A, B)**. Still shots of zoomed in fluorescent microscopy images taken from timelapse videos of horizontal cells from *Tg(Cx57icre; Ai14)* animals. Top panel is the overlay of the brightfield and TdTomato fluorescence signal whereas the bottom panel is the TdTomato alone presented as a grayscale in **(A)** and **(B)**. An example of a horizontal cell (yellow arrow) shown in red in top panel and white in bottom panel, extending neurites and contacting neighboring cells across time **(A)**. Another example of multiple horizontal cells (yellow arrows) forming contacts with one another and to other retinal neurons **(B)**. Images taken from Supplementary Videos. Scale bar = 10 μm.

Next, we performed live imaging to capture the dynamic cellular interactions between horizontal cells and other retinal neurons in culture. Images were captured every 15 min for 48 h from *Tg(Cx57icre; Ai14)* retinas isolated at P8 (Supplementary Videos S1, S2). Still shots of zoomed in regions from the time-lapse videos are shown in Figure 2. The first example is of a single horizontal cell depicted by yellow arrow (red in top panel and white in bottom panel) that begins to extend neurites around 22 h in culture and continues to 48 h in culture, with many of these processes making contacts to neighboring TdTomato-negative neurons as shown in Figure 2A. The other example is of multiple horizontal cells (yellow arrows; red in top panel and white in bottom panel) that begin to contact one another around 13 h, with several processes also contacting neighboring TdTomato-negative neurons (Figure 2B). Timelapse images show horizontal cells are continuously extending neurites and making contacts to different neighboring neurons including other horizontal cells.

### 3.3 Horizontal cells from young animals preferentially extend neurites *in vitro* compared to adolescent or young adult animals

**Figure 3:**
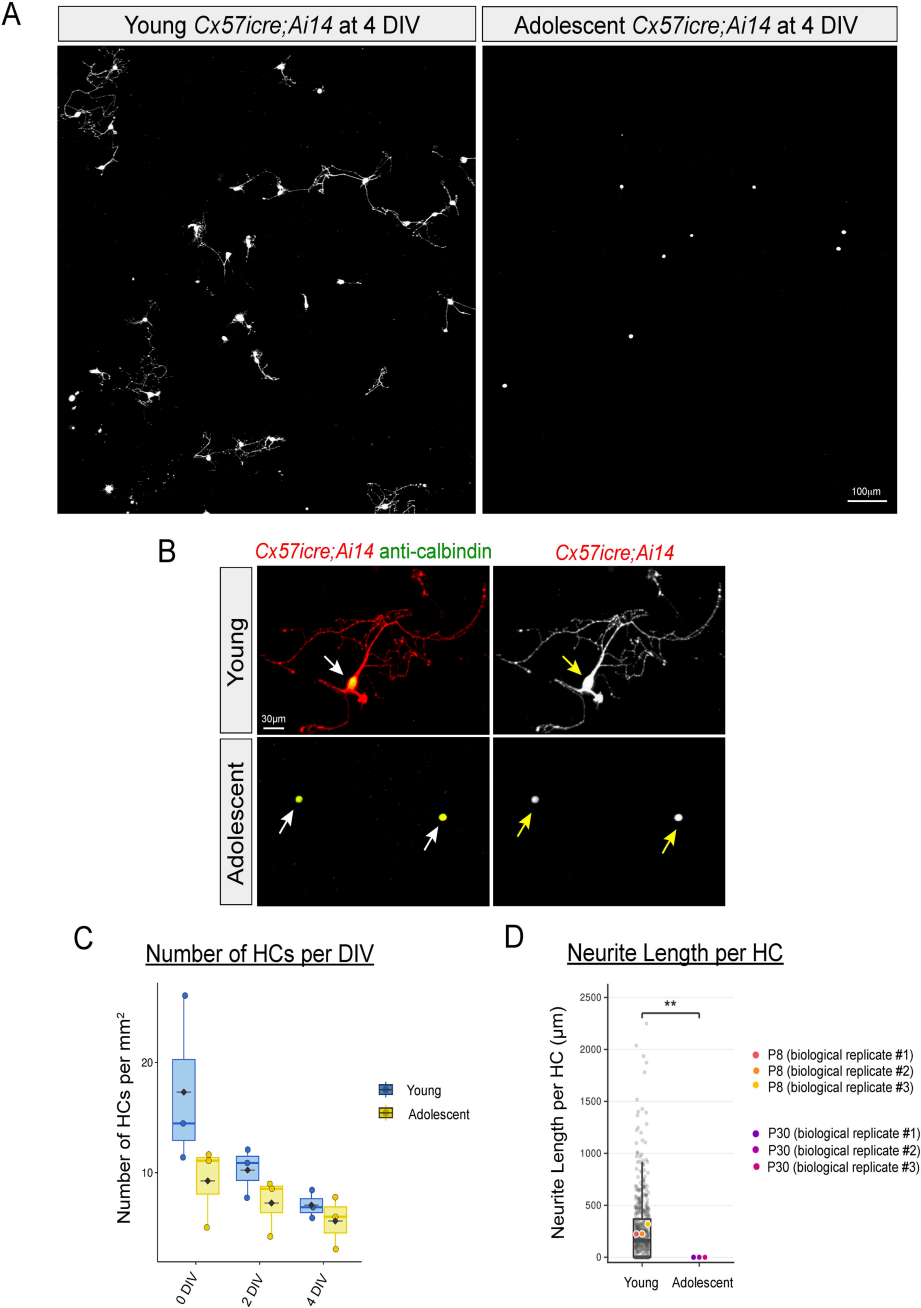
Horizontal cells from young animals but not adolescents extend neurites in culture **(A–D)**. Retinas from *Tg(Cx57icre; Ai14)* animals at P8 (referred to as “Young”) and P30 (referred to as “Adolescent”) were dissociated and cultured for 4 DIV. After 4 DIV, cells were fixed and stained with anti-calbindin. Labeled horizontal cells (+TdTomato) are shown in white from young and adolescent retinas **(A)**. Zoomed in images of horizontal cells from *Cx57icre; Ai14* (red, +TdTomato) stained with anti-calbindin (green) from young and adolescent cultures are shown in **(B)**. Horizontal cells from young retinas but not from adolescent animals extend neurites **(C)**. The average number of horizontal cells (HCs) per DIV of young and adolescent cultures are shown in (C). Mixed-effects modeling detected a significant main effect of DIV but no significant AgeGroup × DIV interaction and no significant P8–P30 differences at any single DIV after Holm correction (all *p* > 0.38). Boxplots with semi-transparent coloration and jittered points show individual biological replicates and the distribution within each age and time point. Average neurite length per HC at 4 DIV in young (P8) and adolescent (P30) cultures are shown in **(D)**. Biological replicate means (*n* = 3 per age) are shown as colored points overlaid on well-level technical measurements (gray). Adolescent (P30) cultures exhibited complete absence of neurite outgrowth at 4 DIV (all values = 0) compared to young (P8) cultures. To account for zero-inflation and non-normality, biological replicate means were log-transformed and compared using Welch’s *t*-test. Young (P8) cultures showed significant neurite outgrowth compared to adolescent (P30) cultures [Welch’s *t*(2) = 11.575, *p* = 0.0037], corresponding to an ~8.1-fold increase (95% CI: 3.72 × −17.66×). **p < 0.01. Scale bar shown on each figure.

We then asked if neurite extension from horizontal cells in culture was restricted to early developmental stages or does this ability continue in the adolescent retina. To answer this question, we isolated and dissociated retinas from *Cx57icre; Ai14* at P8 similar to our prior experiments. We refer to these cultures as “Young.” We also isolated horizontal cells from *Cx57icre; Ai14* mice at adolescent stages or P30 (referred to as “Adolescent”). See Figures 3A, B. A total of three independent biological replicates were used for quantification of each age. At 0 DIV, young cultures at P8 exhibited substantially higher HC counts compared to adolescent cultures at P30 (17.30 *vs.* 9.25 HCs/mm^2^), but this difference narrowed at 2 DIV (10.23 *vs.* 7.24 HCs/mm^2^) and 4 DIV (7.06 *vs.* 5.61 HCs/mm^2^) as shown in Figure 3C. Next, we performed antibody staining at 4 DIV and only both TdTomato-positive and calbindin-positive were used for further analysis. We found horizontal cells isolated from young retinas but not from adolescents extend neurites as shown in Figure 3D. Moreover, neurite outgrowth of young cultures was similar across different technical and biological replicates as shown in Supplementary Figure S2. Our findings demonstrate that horizontal cells from young animals but not those from adolescent stages extend neurites in culture.

### 3.4 Horizontal cells require other retinal neurons to extend neurites in culture

**Figure 4:**
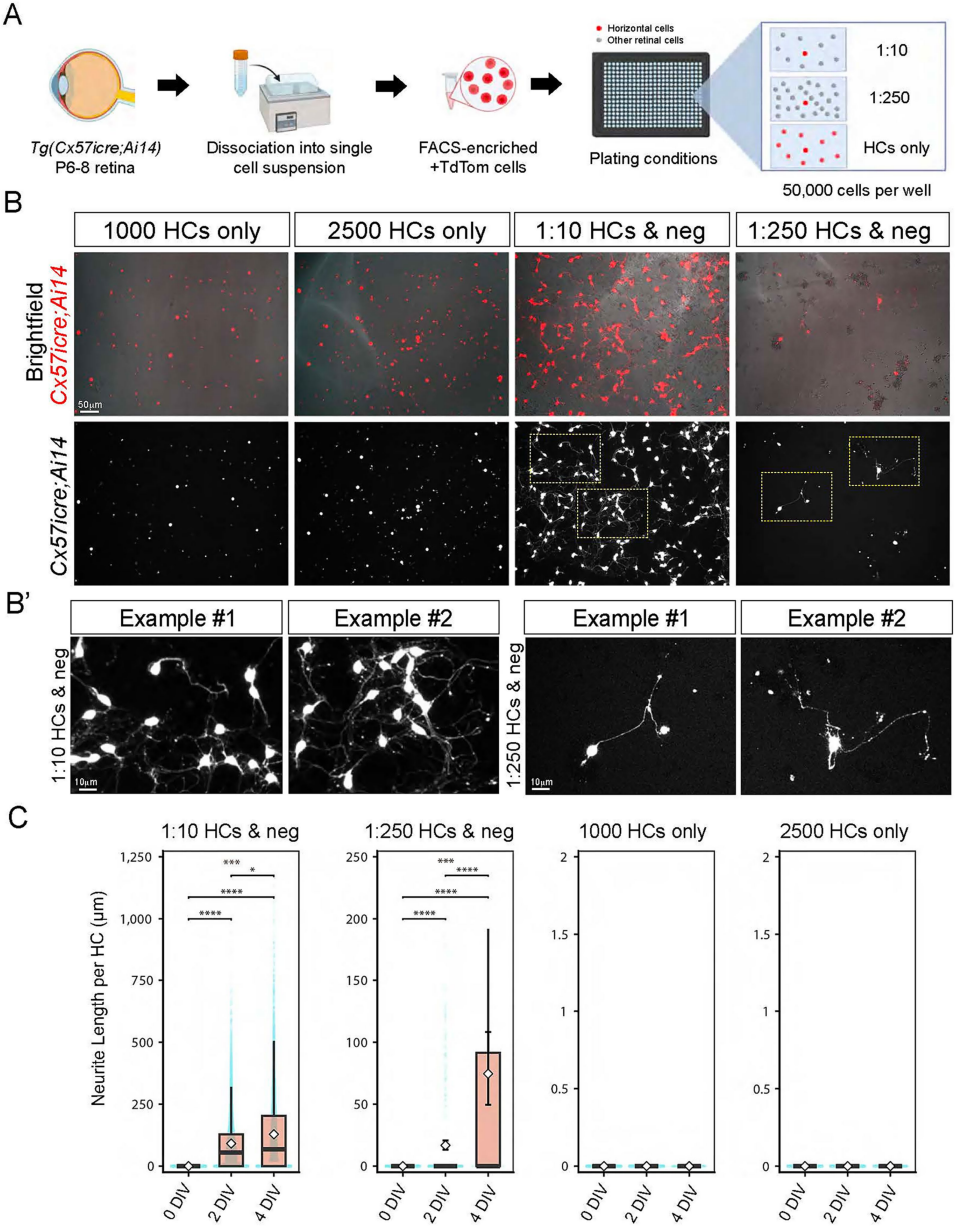
Horizontal cells require other retinal neurons to extend neurites in culture **(A–C)**. Schematic drawing of the experimental design. Horizontal cells (+TdTomato) and other retinal neurons (−TdTomato) were isolated from *Tg(Cx57icre; Ai14)* animals via FACS. Horizontal cells (HCs) or +TdTomato cells were either cultured alone at 1000 HCs or 2,500 HCs or at different ratios (1:10, 1:25) with other -TdTomato or negative (neg) cells. Horizontal cells are shown in red on the top panel which is an overlay of the bright field and TdTomato fluorescence signal **(B)**. The bottom panel is the TdTomato fluorescence presented as a grayscale where horizontal cells are shown in white **(B)**. Horizontal cells cultured for 4 DIV display distinct morphology in the various conditions **(B)**. Horizontal cells plated alone at 1000 cells or 2,500 cells per well showed no neurite outgrowth in **(B)**. However, horizontal cells plated with other retinal neurons (−TdTom) at a ratio of 1:10 or 1:250 showed extensive neurite outgrowth **(B)**. Insets in **(B’)** are zoomed images of HCs with neurites (yellow boxes) in the 1:10 and 1:250 HCs and neg condition in **(B)**. Neurite length per HC was measured at 0, 2, and 4 DIV across the different co-culture conditions in **(C)**. Individual cell measurements are shown as scatter points, with boxplots indicating medians and quartiles; white diamonds represent means with 95% bootstrap confidence intervals. Both 1:10 and 1:250 HC and neg showed highly significant changes across DIV (Kruskal–Wallis *p* < 1 × 10^−78^). Post-hoc Dunn tests (Benjamini–Hochberg corrected) revealed that for the 1:10 HC and neg condition, neurite length increased significantly from 0 to 2 DIV and from 0 to 4 DIV (*p* = 0 for both), with a modest but significant increase between 2 and 4 DIV (*p* = 0.042). For the 1:250 HC & neg condition, all time-point comparisons were strongly significant (0 *vs.* 2 DIV: *p* = 5.21 × 10^−40^; 0 *vs.* 4 DIV: *p* = 9.39 × 10^−53^; 2 *vs.* 4 DIV: *p* = 4.25 × 10^−26^). In contrast, HC cultured alone at 1000 or 2,500 HCs per well exhibited no neurite outgrowth at any time point (all measurements = 0). Because these distributions contained only zeros, no statistical tests were applicable for these conditions. Data were zero-inflated and right-skewed; therefore, nonparametric tests were used. Geometric mean neurite lengths for co-culture conditions increased consistently over time [e.g., 2 DIV: 88.3 μm (95% CI: 86.3–90.2); 4 DIV: 153.6 μm (149.6–157.6)]. ****p < 0.0001, ***p < 0.001, *p < 0.05. Scale bar shown on figure.

Next, we addressed whether horizontal cells in culture require other neuronal types to promote neurite outgrowth. To test this, we isolated horizontal cells via Fluorescence-activated cell sorting (FACS) from *Cx57icre; Ai14* animals at P8 and plated them in different conditions as shown in Figure 4A. For each experiment, we used six retinas from three different transgenic animals and a total of 5–6 technical replicates were used for quantification per experimental condition. We gated for single, viable cells and collected TdTomato-positive (+TdTom) and TdTomato-negative (−TdTom) fluorescently labeled cells. We then plated horizontal cells (+TdTom) either alone or with other viable retinal neurons (−TdTom) at different ratios as shown in Figure 4A in order to determine the appropriate ratio of +TdTom to -TdTom cells to maximize neurite outgrowth of horizontal cells. We then tracked overall cell number and neurite length per cell across days *in vitro* or DIV. We found FACS-isolated horizontal cells plated alone at either 1,000 or 2,500 cells per well did not extend neurites after 4 DIV (Figure 4B). However, the same FACS-isolated horizontal cells co-cultured with other retinal neurons at a 1:10 or 1:250 ratio showed extensive neurite outgrowth (Figure 4B). Zoomed in images of horizontal cells plated at a 1:10 or 1:250 ratio with other retinal neurons showed robust neurite outgrowth at 4 DIV as shown in Figure 4B’. We quantified these observations by measuring the neurite length per HC across DIVs in the different co-culture conditions (Figure 4C). Our data showed horizontal cells cultured with other retinal neurons at a 1:10 ratio had significant neurite outgrowth from 0 to 4 DIV compared to HCs cultured alone that never extended neurites. Moreover, HCs cultured with other retinal neurons at a 1:10 ratio survived better across DIVs compared to those HCs plated alone (Supplementary Figure S3). Interestingly, HCs cultured at a 1:250 ratio with other retinal neurons showed reduced cell counts from 0 to 4 DIV as shown in Supplementary Figure S3 but displayed significant neurite outgrowth from 0 to 4 DIV (Figure 4C). These results show there is significant neurite outgrowth of FACs-isolated horizontal cells in culture when plated with other retinal neuron types and not when cultured alone.

### 3.5 Retinal ganglion cells (RGCs) but not horizontal cells (HCs) extend neurites in a co-culture

**Figure 5:**
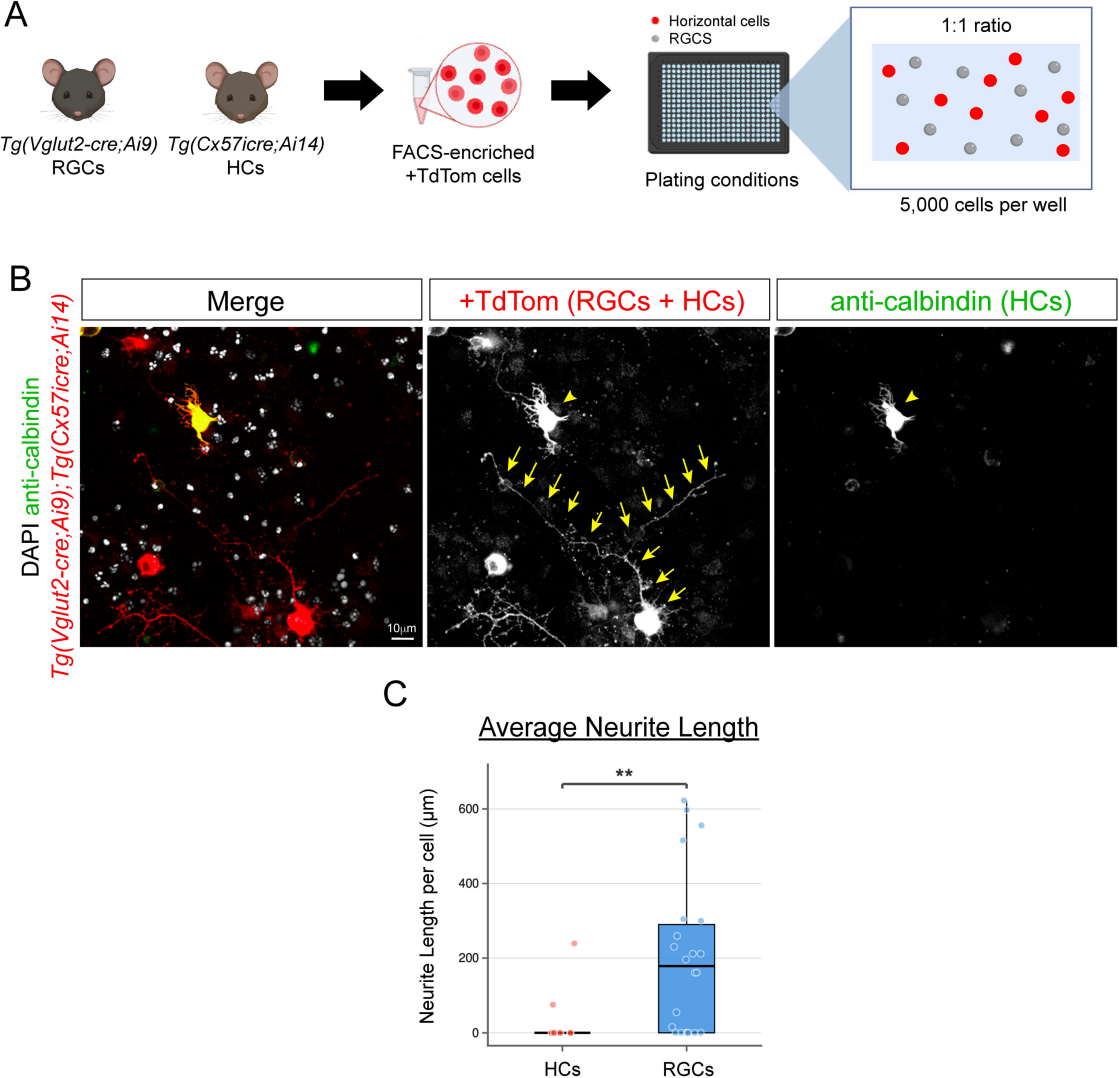
Retinal ganglion cells (RGCs) but not horizontal cells (HCs) extend neurites in a co-culture **(A–C)**. Schematic drawing of the co-culture experimental approach **(A)**. Cells were fixed and stained with anti-calbindin (green) after 4 DIV to distinguish between RGCs and HCs. RGCs were TdTomato-positive and calbindin-negative, whereas HCs were TdTomato-positive and calbindin-positive in **(B)**. RGCs extend long neurites as depicted by yellow arrows compared to horizontal cells (yellow arrowhead) in a co-culture as shown in **(B)**. Quantification of the neurite length per cell for both RGCs and HCs at 4 DIV is shown in **(C)**. HCs were isolated from three *Tg(Cx57icre; Ai14)* animals (*n* = 11 from 6 retinas) and RGCs were isolated from three *Tg(Vglut2-cre; Ai9)* animals (*n* = 22 from 6 retinas). Individual cells are shown as jittered points with boxplots depicting medians and quartiles. Nearly 82% or 9 out of 11 HCs failed to extend neurites after 4 DIV, whereas only 32% or 7 out of 22 of the RGCs did not extend neurites. This difference was significant as determined by a Fisher’s exact test (*p* = 0.010). A Mann–Whitney *U* test on all values detected a significant overall difference between the two groups (W = 55.5, *p* = 0.0084). Geometric mean estimates reflect this disparity in zero inflation: HCs = 134.25 μμm (95% CI: 0.09–205,077 μm; only 2 nonzero values), RGCs = 217.41 μm (95% CI: 127.55–370.57 μm; *n* = 15 nonzero). **p < 0.01. Scale bar shown on image.

Retinal ganglion cells (RGCs) have been well-documented to extend neurites even when cultured alone (Park et al., 2020; Kerrison and Zack, 2007). Thereby, we investigated if RGCs could promote neurite outgrowth of horizontal cells in a co-culture system. To test this possibility, we FACs-isolated horizontal cells from three different *Tg(Cx57icre; Ai14)* animals similar to our previous experiments, and FACs-isolated separately RGCs from three different *Tg(Vglut2-cre; Ai9)* animals as illustrated in Figure 5A. We plated a total of 5,000 cells per well at a 1:1 ratio of RGCs to HCs (see Figure 5A). After 4 DIV, we fixed the cells and performed antibody staining with anti-calbindin to distinguish between RGCs and HCs. Cells that were TdTomato-positive and calbindin-positive were the HCs, whereas those that were TdTomato-positive and calbindin-negative were the RGCs (Figure 5B). Next, we measured neurite outgrowth at 4 DIV and found the majority of HCs failed to extend neurites (82% with zero measurements) whereas RGCs only showed 32% with zero values (Figure 5C). Moreover, RGCs extended longer neurites compared to HCs in co-culture as shown in Figure 5C. These findings demonstrate that horizontal cells but not RGCs require other retinal neurons for neurite outgrowth in culture. This is not surprising as HCs and RGCs do not form direct synaptic contacts within the mouse retina. The outgrowth of HCs in culture may require specific contact-mediated cues for their respective synaptic partners (i.e., photoreceptors) and not from other neuronal cell types.

## 4 Discussion

In summary, we present a cell culture system that can be used to study the mechanisms underlying horizontal cell specificity during retinal development. We utilized the *Tg(Cx57icre)* mouse line which is known to selectively target horizonal cells (Hirano et al., 2016). Although there have been several studies on retinal cell cultures (Zhang et al., 2002; Ogilvie et al., 1999; Romano and Hicks, 2007; Schnichels et al., 2021) including work on horizontal cells from different species (Jellali et al., 2002; Hidaka et al., 1989; Withrow and Johnson, 2001; Schubert et al., 2010), to our knowledge, this is the first that uses a transgenic mouse culture system to visualize and quantify neurite outgrowth of horizontal cells. We found horizontal cells in culture extend multiple and complex processes similar to what is seen *in vivo* across development. However, this was largely dependent on other retinal neurons being present in culture as the same number of horizontal cells cultured alone failed to extend neurites. We also found that horizontal cells cultured from young animals (P8) preferentially extended neurites compared to adolescents (P30). These data suggest that there must be developmental mechanisms that promote neurite outgrowth of horizontal cells at early stages, and these are no longer present in the adolescent, suggesting some type of plasticity that occurs during younger developmental time points. Taken together, we demonstrate how an *in vitro* system could be used to decipher the complex cellular and molecular mechanisms involved in neural circuit assembly.

Horizontal cells are known to synapse selectively to the distinct types of photoreceptors via different subcellular compartments. The dendrites of horizontal cells synapse to cone photoreceptors whereas the axon terminal connects to rod photoreceptors (Kolb, 1974). During development, photoreceptors are known to play an important role in horizontal cell morphology. Work by Reese and colleagues showed that the type of photoreceptors in the retina directly influences dendritic and axonal morphology of horizontal cells (Raven et al., 2007). Retinas where rods have been converted to cones though disruption of the rod-specific transcription factor *Nrl* (referred to as “cone-full”) results in more processes in the dendrites of horizontal cells and less in the axon terminal compared to controls. Conversely, ectopic expression of *Nrl* leads to cones being converted to rods (referred to as “rod-full”), and horizontal cells display less processes in the dendrites and more in the axon terminal. These data demonstrate that there must be signaling mechanisms between photoreceptors and horizontal cells that promote neurite outgrowth of the different cellular compartments (i.e., dendrites or axon terminal) during development. Our culture system of horizontal cells could be used to further study these cellular mechanisms in the developing retina using our live-cell imaging techniques.

Our data shows horizontal cells from both young and adolescent retinas retain the ability to extend neurites in culture; however, the average neurite length per horizontal cell is significantly lower in adolescents compared to young animals. These data suggest that there must be signaling factors that promote neurite outgrowth during development, and these are either repressed or no longer present in the adolescent animal. Work by Soto and colleagues support this model as re-introduction or overexpression of the cell adhesion molecule, NGL2 in the adolescent mouse retina is sufficient to restore and promote neurite outgrowth of horizontal cells *in vivo* (Soto et al., 2018). Moreover, recent advancements in single neuron labeling approaches have allowed us to visualize horizontal cells at distinct developmental stages (Veldman et al., 2020). As the retina develops in a central-to-peripheral wave (Rapaport et al., 2004; Young, 1985), horizontal cells located in the periphery tend to have multiple axons whereas those located in the center have only one bona fide axon similar to what is seen in the adolescent retina (Veldman et al., 2020). This suggests there must be neurite remodeling that occurs at early developmental stages that ultimately lead to the stereotypic morphology of horizontal cells observed in the mature retina.

Published studies have shown only certain retinal neuron types can extend neurites in culture. For instance, RGCs from both young and adolescent retinas are known to extend neurites in culture (Park et al., 2020; Kerrison and Zack, 2007; Lilley and Robbins, 2005), whereas photoreceptors show minimal neurite outgrowth and poor survival in culture (Sarin et al., 2018; Zhu et al., 2022). We found horizontal cells can extend complex and elaborate processes similar to *in vivo*; however, this ability is impaired when horizontal cells are plated alone or in older animals. This raises the question of what could be the factors that promote neurite outgrowth of horizontal cells at early developmental stages. Recent data has shown rod photoreceptors secrete trophic factors such as Rod-derived cone viability factor (RdCVF) to promote cone photoreceptor survival (Ait-Ali et al., 2015; Leveillard et al., 2004; Ait-Ali and Leveillard, 2022). Similarly, photoreceptors which are known to synapse to horizontal cells could secrete specific growth factors to promote neurite outgrowth during development. However, this fails to address why certain neurons in culture retain the ability to extend neurites in culture, whereas others are limited to their *in vivo* environment. Identifying these factors may be key to devising new forms of therapies that can promote regeneration of different cell types in retinal diseases.

The present study was conducted utilizing an *in vitro* culture system of horizontal cells that permitted analysis and quantification of neurite processes at specific time points. While this system provides certain advantages, it is important to recognize that there are certain limitations. First, certain features of the *in vivo* retinal environment are not present here such as the absence of glial cells (i.e., Muller glia) and the retinal vasculature. Second, recent studies have shown P30 in mice is considered young adults or adolescents (Arellano et al., 2024), suggesting there could still be some level of neuroplasticity. Thus, examining neurite outgrowth of horizontal cells at later stages would be interesting for future studies. Third, both males and females were used indiscriminately in all experiments. However, there could be sex-specific differences in horizontal cell development. And lastly, increasing the number of biological replicates would optimize the study and allow for more robust statistical analyses of the data distribution. Moreover, we noticed cultures that were derived from six retinas or three animals versus those from two retinas or one animal appeared healthier with more robust neurite outgrowth (data not shown). This could be due to the presence of more neurons especially those that secrete growth factors needed for cell survival and neurite outgrowth of horizontal cells. Additional studies will be required to identify these essential growth factors.

In conclusion, our data demonstrates horizontal cells extend neurites in a temporal- and cellular-dependent manner in culture similar to what is seen *in vivo*. Future studies are needed to address the cellular and molecular mechanisms responsible for synaptic specificity of horizontal cells during retinal development. These studies are critical as they will reveal general principles underlying circuit assembly in the developing central nervous system.

## Data Availability

The original contributions presented in the study are included in the article/Supplementary material, further inquiries can be directed to the corresponding author.

## Supplementary material

SUPPLEMENTARY FIGURE 1 Confocal image of a mixed retinal cell culture at 4 DIV isolated from *Tg(Cx57icre;Ai14)* animals at P8. Cultures were stained with anti-calbindin (magenta), anti-Rhodopsin (green), and DAPI to label horizontal cells and rod photoreceptors in **(A)**. A single horizontal cell depicted by yellow arrow shows labeling with anti-calbindin but not anti-Rhodopsin. Two examples of Rhodopsin-positive cells are provided in **(A’)**. Insets are zoomed regions of yellow boxed areas in **(A)**.

SUPPLEMENTARY FIGURE 2 Violin plots illustrate how the average neurite length for each horizontal cell (HC) per culture is highly reproducible between technical and biological replicates in young animals. A total of three independent biological replicates with three technical replicates for each are shown on graph. The internal dashed lines indicate the median and interquartile range. Statistical analysis was performed on the three independent biological replicates as shown in Figure 1D.

SUPPLEMENTARY FIGURE 3 The number of HCs were quantified at 0, 2, and 4 DIV across the four co-culture experimental conditions. Boxplots display biological replicate means, with jittered points representing individual replicates, and technical replicates with grey points. To determine DIV-dependent changes within each experimental condition, paired t-tests with Holm correction were applied to biological replicate means. For conditions with only two biological replicates, statistical outcomes are presented with caution due to limited power. Significant differences after Holm adjustment (*p* < 0.05) are indicated by asterisks. **p* < 0.05; ns, not significant.

SUPPLEMENTARY VIDEO 1 Timelapse imaging of a mixed retinal culture isolated from P8 *Tg(Cx57icre;Ai14)* animals. Horizontal cells express TdTomato and are shown in red in the video. Images were acquired for a period of 48 hours at a speed of 97 frames with 30 minutes between frames. Still shots and zoomed in images are presented in the main figure (Figure 2A). Yellow arrow indicates cell of interest. A cell that moves out of focused is indicated by an asterisk.

SUPPLEMENTARY VIDEO 2 Another example of a time-lapse movie showing neurite extensions of multiple horizontal cells (shown in red) depicted by yellow arrows from *Tg(Cx57icre;Ai14)* mice isolated at P8. Images were acquired at a speed of 97 frames, 30 mins between frames for 48 hours. Zoomed in images are shown in the main figure (Figure 2B). Asterisk denotes a cell that moves out of frame.
