# Supplementary figures and images for: Utilizing a culture system for horizontal cells to study neural circuit assembly in the developing mouse retina
Source: Front Cell Neurosci. 2026 Apr 1;20:1691122. doi: 10.3389/fncel.2026.1691122 (PMC13079198; doi:10.3389/fncel.2026.1691122)

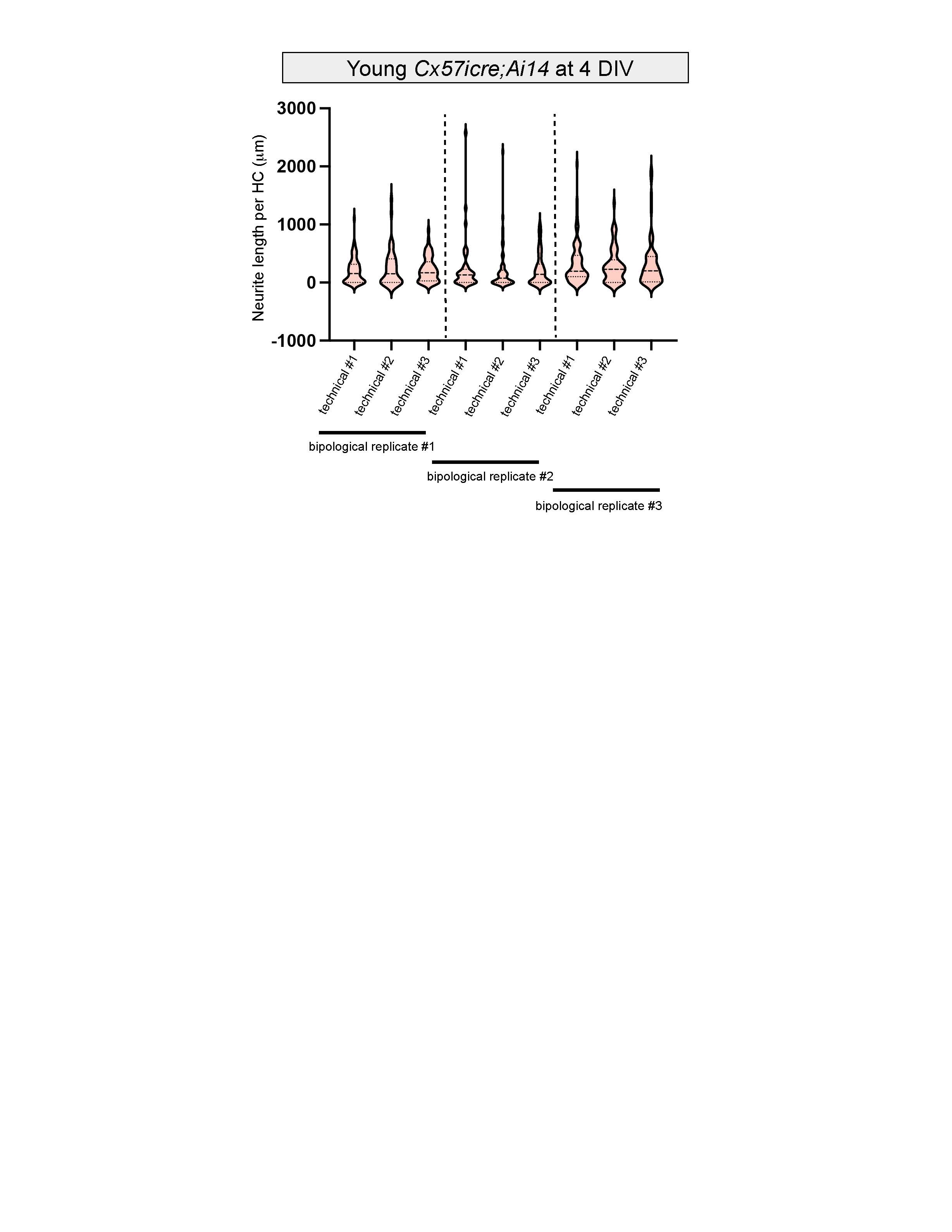

Supplement: SUPPLEMENTARY FIGURE 2 — Violin plots illustrate how the average neurite length for each horizontal cell (HC) per culture is highly reproducible between technical and biological replicates in young animals. A total of three independent biological replicates with three technical replicates for each are shown on graph. The internal dashed lines indicate the median and interquartile range. Statistical analysis was performed on the three independent biological replicates as shown in Figure 1D. [file Image_2.JPEG]

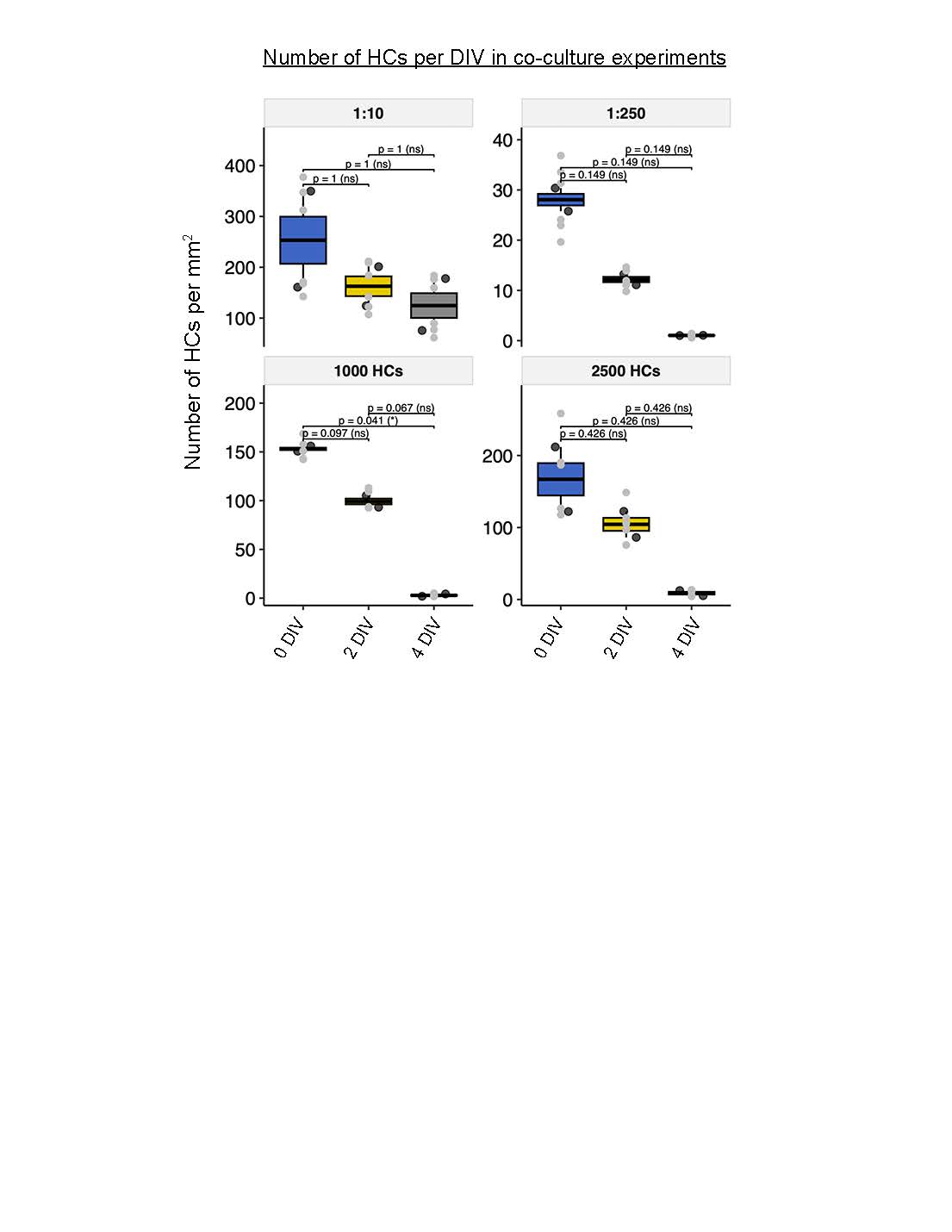

Supplement: SUPPLEMENTARY FIGURE 3 — The number of HCs were quantified at 0, 2, and 4 DIV across the four co-culture experimental conditions. Boxplots display biological replicate means, with jittered points representing individual replicates, and technical replicates with grey points. To determine DIV-dependent changes within each experimental condition, paired t-tests with Holm correction were applied to biological replicate means. For conditions with only two biological replicates, statistical outcomes are presented with caution due to limited power. Significant differences after Holm adjustment (p < 0.05) are indicated by asterisks. *p < 0.05; ns, not significant. [file Image_3.JPEG]
